# Role of Gut‐Derived Endotoxins in Porto‐Sinusoidal Vascular Disorder: Comparison Between patients with and without portal hypertension

**DOI:** 10.1111/liv.70277

**Published:** 2025-08-08

**Authors:** Diletta Overi, Nicola Pugliese, Roberto Carnevale, Silvia Nardelli, Daniele Tavano, Lorenzo Ridola, Alessandro Mari, Manuela Merli, Luca di Tommaso, Luigi Terracciano, Giulia d'Amati, Andrea Baiocchini, Maurizio Forte, Giacomo Frati, Guido Carpino, Alessio Aghemo, Oliviero Riggio, Eugenio Gaudio, Stefania Gioia

**Affiliations:** ^1^ Department of Anatomical, Histological, Forensic Medicine and Orthopedic Sciences Sapienza University of Rome Rome Italy; ^2^ Division of Internal Medicine and Hepatology, Department of Gastroenterology IRCCS Humanitas Research Hospital Rozzano Italy; ^3^ Department of Biomedical Sciences Humanitas University Pieve Emanuele Italy; ^4^ Department of Medical‐Surgical Sciences and Biotechnologies Sapienza University of Rome Latina Italy; ^5^ IRCCS Neuromed Pozzilli Italy; ^6^ Department of Translational and Precision Medicine Sapienza University of Rome Rome Italy; ^7^ Pathology Unit IRCCS Humanitas Research Hospital Rozzano MI Italy; ^8^ Department of Radiological, Oncological, and Pathological Sciences Sapienza University of Rome Rome Italy; ^9^ Department of Pathology San Camillo Forlanini Hospital Rome Italy

**Keywords:** endotoxemia, intestinal permeability, obliterative portal venopathy, porto‐sinusoidal vascular liver disorder

## Abstract

**Background:**

Etiopathogenesis of porto‐sinusoidal vascular disorder (PSVD) is poorly known. The present study aimed to investigate alterations in gut barrier, bacterial translocation, and pro‐aggregating/pro‐coagulant state and their relationship with liver injury in patients with PSVD without portal hypertension (PH−) in comparison with PSVD with PH (PH+) and healthy controls.

**Methods:**

34 patients with PSVD (17 PH+ and 17 PH−) and 17 healthy subjects were submitted to measurement of zonulin and lipopolysaccharides (LPS), markers of intestinal permeability, of s‐Glycoprotein VI, sP‐selectin, ADAMTS13 and von Willebrand factor (vWF), markers of platelet aggregation and vascular dysfunction, factor VIII and F1 + 2, markers of hypercoagulability. In 30 PSVD patients, a histomorphological/immunohistochemical study on liver biopsies was performed.

**Results:**

PSVD PH− patients had higher serum levels of LPS, zonulin, vWF, factor VIII, sP‐selectin, F1 + 2 and lower levels of ADAMTS13 compared to healthy controls. These alterations were even more pronounced in PSVD PH+. At histological analysis, compared to those of healthy subjects, livers of patients with PSVD PH− showed a higher number of TLR4+ macrophages and of platelets within sinusoids with signs of aggregation. Perivascular fibrosis and sinusoid capillarisation were higher too. PSVD PH− had a lower degree of obliterative portal venopathy and portal inflammation compared to patients PH+.

**Conclusions:**

Even before the development of PH, patients with PSVD exhibit increased intestinal permeability and bacterial translocation, related platelet aggregation, and hypercoagulability, suggesting that endotoxemia may play a pivotal role in the pathogenesis of vascular alterations underlying PSVD. Moreover, this study indicates that PSVD without and with PH represent different stages of the same disease.

**Summary**

- Patients with a histological diagnosis of PSVD and without portal hypertension (PH) already exhibit alterations in gut barrier integrity, increased translocation of bacteria‐derived particles, and related consequences such as platelet aggregation and hypercoagulability.
- These findings suggest that endotoxemia could play a key role in the pathogenesis of vascular alterations underlying PSVD.
- PSVD without and with PH define the same disease but at different stages, where PSVD without PH could represent an early stage of the disease that may progress to a clinically more advanced stage represented by PSVD with PH.

**Abbreviations**
CECT: contrast‐enhanced computed tomography
EDTA: vWF von Willebrand factor
F1 + 2: prothrombin fragment F1 + 2
H&E: haematoxylin and eosin
LPS: lipopolysaccharides
OPV: obliterative portal venopathy
PBS: phosphate‐buffered saline
PH: portal hypertension
PLT: platelets
PSVD: porto‐sinusoidal vascular liver disorder
PT: portal tracts
RT: room temperature
sGPVI: soluble glycoprotein VI
sPs: soluble p‐selectin
SR: sirius red
TLR4: toll‐like receptor 4

## 1 Introduction

Porto‐sinusoidal vascular disorder (PSVD) is a vascular liver disease characterised by histo‐morphologic changes in the liver affecting the interlobular branches of the portal vein and the sinusoids, often resulting in the development of portal hypertension (PH) and rarely progressing to liver failure requiring liver transplantation [1, 2, 3]. In a minority of patients, typical histological lesions (e.g., obliterative portal venopathy and incomplete septal fibrosis) are not associated with clinically evident portal hypertension [4, 5, 6]. Immune disorders, gastrointestinal disorders, and abdominal infections are frequently associated with PSVD [7, 8, 9] and may play a role in the development of the disease. A possible role of the gut and intestinal infections is supported by experimental evidence that intraportal injection of killed non‐pathogenic *Escherichia coli* in rabbits and dogs leads to the development of PH and of histological lesions of the liver similar to those observed in PSVD [10, 11, 12]. In addition, a rat model of PSVD has recently been developed through the infusion of a bolus of 1 mg/kg of lipopolysaccharides (LPS) derived from *Escherichia coli* (O111:B4) into the portal vein, administered twice weekly for 8 weeks. This model resulted in the development of PH, with liver histology revealing typical PSVD lesions in the absence of cirrhosis [13].

In a recent study, we showed that patients with PSVD and PH had an alteration of the integrity of the intestinal barrier with bacterial translocation into the systemic circulation [14]. Moreover, the binding of LPS with TLR4 + ‐macrophages, highly expressed in the liver of PSVD patients, was associated with the presence of endothelial damage, with a pro‐aggregating and pro‐coagulant state as well as with liver fibrosis, portal inflammation, obliterative portal venopathy (OPV) and biliary tree injury. However, as all PSVD patients included in the study had PH, it could not be definitely established if the increased intestinal permeability and the consequent bacterial translocation observed in these patients were the consequence of PH rather than a cause of PSVD.

The aim of the present study was to evaluate bacterial translocation and the resulting platelet aggregation and hypercoagulability in patients with PSVD without PH and to analyse their relationship with histologic features in comparison with a population of patients with PSVD with PH and healthy controls.

## 2 Patients and Methods

This bicentric prospective study included all patients with PSVD without PH who were referred to the ‘Portal Hypertension’ outpatient clinic at Policlinico Umberto I in Rome and to the Hepatology Unit at Humanitas Research Hospital in Milan between January 2021 and March 2024.

Written informed consent was obtained from each patient, and the study was conducted in accordance with the ethical principles of the Declaration of Helsinki. Patients or the public were not involved in the manuscript. The institutional review board of the Sapienza University of Rome (Prot no. 0407/2022, ref. 6713) and of Humanitas Research Hospital (Prot no. 155/23) approved the study protocol.

Patients aged > 18 and < 80 years with a biopsy‐proven diagnosis of PSVD without clinically significant PH were eligible for inclusion in the study. The diagnosis of PSVD was based on the VALDIG criteria [1]. PH was excluded in these patients based on the absence of clinical signs of PH, as all had undergone upper gastrointestinal endoscopy and contrast‐enhanced computed tomography (CECT) within 6 months of histological diagnosis to rule out oesophageal or gastric varices, portosystemic collaterals, splenomegaly (spleen size ≥ 13 cm in the largest axis) or ascites. All patients underwent regular clinical follow‐up after enrolment, including blood tests and abdominal ultrasound with Doppler every 6 to 12 months to monitor for the potential development of PH.

Exclusion criteria were infection or sepsis and/or ongoing treatment with systemic or non‐absorbable antibiotics, use of aspirin or other non‐steroidal anti‐inflammatory drugs in the previous 30 days, platelets or plasma transfusion in the previous 30 days, pregnancy or breastfeeding, and active solid tumours.

Seventeen patients with PSVD without PH who met the histological criteria of the study were finally enrolled. Patients who did not fulfil the inclusion criteria were not included at all in the study. The indication for liver biopsy in these patients was based on evidence of unexplained and persistent alteration of liver enzymes.

A group of 17 patients with PSVD and clinically evident PH were included as controls. Cases and controls were matched for age and comorbidities, and a 1:1case–control ratio was selected. The diagnosis of PSVD was biopsy‐proven and was based on the VALDIG criteria [1] in all cases. PH was defined by the presence of oesophago‐gastric varices, portal‐hypertensive bleeding, portosystemic collaterals, splenomegaly/hypersplenism, and/or ascites.

All patients with PSVD were submitted to liver stiffness measurements (LSM) by transient elastography (TE) using the Echosens Fibroscan device. LSM was performed by hepatologists trained for TE, and TE‐LSM was considered reliable when IQR/TE‐LSM ≤ 0.30 and > 10 valid measurements.

A group of age‐ and sex‐matched healthy volunteers was included as control, and a case–control ratio of 1:1 was selected.

At the time of enrolment, all the patients were submitted to a peripheral venous blood sample, subsequently centrifuged at 2500×*g* for 10 min. Blood samples were taken from the antecubital vein in all patients, collected in tubes without anticoagulant or with 3.8% sodium citrate, lithium heparin, and EDTA, and centrifuged at 300×*g* for 10 min to obtain the supernatant. All plasma and serum aliquots were stored at −80°C in appropriate cuvettes until assayed. In all cases, blood samples were collected within 3 months of liver biopsy.

Indirect markers of intestinal permeability and of bacterial translocation, such as zonulin and lipopolysaccharide (LPS), respectively, markers of platelet aggregation, such as soluble glycoprotein VI (sGPVI) and soluble p‐selectin (sPs), markers of hypercoagulability status, such as factor VIII and prothrombin fragments F1 + 2, and markers of vascular dysfunction, such as ADAMTS13 and vWF were measured.

### 2.1 Serological Biomarkers Assay

Serum zonulin was used as an intestinal permeability assay. Serum zonulin levels were measured using a commercial ELISA kit (Elabscience, Houston, TX, USA, code number E‐EL‐H5560). Antibody specific for zonulin has been pre‐coated onto a microplate and 100 μL of standards and patient sera samples were added and incubated for 90 min at 37°C. Then, a biotinylated detection antibody specific for zonulin and Avidin‐Horseradish Peroxidase (HRP) conjugate was added to each microplate. Values were expressed as ng/mL; both intra‐assay and inter‐assay coefficients of variation were within 10%.

Endotoxin (lipopolysaccharide, LPS) serum levels were measured using a commercial ELISA kit (Cusabio, Maryland, USA, code number CSB‐E09945h). Standards of LPS, purified from *Escherichia coli* , or samples were plated for 2 h at room temperature onto a microplate pre‐coated with the antibody specific for LPS. After incubation, samples were read at 450 nm. Values were expressed as pg/mL; intra‐assay and inter‐assay coefficients of variation were 8% and 10%, respectively.

Quantification of soluble Glycoprotein VI (sGPVI) was performed in plasma samples by a validated enzyme immunoassay method (Thermo Fisher Scientific, Waltham, Massachusetts, USA, code number EH230RB). Values were expressed as pg/mL. Intra‐assay and inter‐assay coefficients of variation were < 10% and < 12%, respectively.

Soluble P‐selectin (sP‐selectin, Thermo Fisher Scientific, Waltham, Massachusetts, USA, code number BMS219‐4) levels were measured with a commercial immunoassay. Intra‐ and inter‐assay coefficients of variation were 4.3% and 6.1% respectively. The values were expressed in ng/mL for sP‐selectin.

Factor VIII (Lifespan Bioscience, Seattle, Washington, USA, code number LS‐F10415e) was measured by ELISA method according to the manufacturer's guidelines. The values of plasma of factor VIII were expressed as U/dL. Inter‐ and intra‐assay coefficient of variations were < 10%.

Plasma levels of human prothrombin fragment F1 + 2 (F1 + 2) were assayed by an enzyme immunoassay based on the sandwich principle (Cusabio, Maryland, USA, code number CSB‐E09822h). The values of plasma F1 + 2 were expressed as pmol/L. Intra‐assay and inter‐assay coefficients of variation for this method were < 8% and < 10%, respectively.

ADAMTS13 antigen was measured using VWFCP/ADAMTS13 ELISA kits (Elabscience, Houston, TX, USA, code number E‐EL‐H1552). Values were expressed as ng/mL. Intra‐assay and inter‐assay coefficients of variation were < 10% respectively.

The von Willebrand factor (vWF, Abnova, Taipei City, Taiwan, code number KA0512) was measured by ELISA according to the manufacturer's guidelines. The plasma values of vWF were expressed as U/dL. Inter‐ and intra‐assay coefficients of variation were < 10%.

### 2.2 Histomorphology and Immunohistochemistry on Liver Biopsies

Liver biopsy specimens were available for all included PSVD patients, with and without PH (*N* = 34). As histologic controls, *N* = 6 liver samples from liver donors were used [15].

Three‐μm sections were obtained from the pathology department. Histomorphological stains (i.e., haematoxylin and eosin—H&E and sirius red—SR) were performed according to standard protocols. For immunohistochemistry, endogenous peroxidase activity was blocked by a 20‐min incubation in hydrogen peroxide (4%). Antigens were retrieved, as indicated by the vendor, by applying Proteinase K (Dako, Glostrup, Denmark, code S3020) for 10 min at room temperature. Sections were incubated overnight at 4°C with primary antibodies (Table S1). Then, samples were rinsed twice with phosphate buffered saline (PBS) for 5 min, incubated for 30 min at room temperature (RT) with dextran‐HRP conjugated secondary antibody (REAL EnVision Detection System Peroxidase, Dako, Glostrup, Denmark code K5007). Diaminobenzidine (Dako, Glostrup, Denmark code K3468) was used as substrate, and sections were counterstained with haematoxylin.

Histological sections were analysed in a coded fashion by two independent researchers using a light microscope (Leica Microsystems DM4500B; Leica Microsystems, Weltzlar, Germany) equipped with a Leica K3C Videocam and LAS X Core software. Slides were further scanned by a digital scanner (Aperio ScanScope CS and FL Systems; Aperio Digital Pathology, Leica Biosystems, Milan, Italy) and processed by ImageScope. In cases of discordance between the two observers, a collegial review of the case was performed on digitalised slides, and an agreement was reached for all evaluations.

A histomorphological study of slides was performed by evaluating specific and non‐specific histological signs of PSVD [1, 2, 3]. The number of portal tracts with OPV was counted on the whole slide and is expressed as a percentage of affected portal tracts. For other histological features (i.e., sinusoidal dilatation, portal inflammation, perisinusoidal fibrosis, portal fibrosis and septal fibrosis), a semi‐quantitative score was used to grade the severity of the lesions by modifying scoring systems adopted for other liver diseases [14, 16], as reported in Table S2.

Sinusoidal capillarisation was evaluated on CD34‐stained slides, and the percentage of the liver lobule occupied by CD34+ sinusoids was calculated by an image analysis algorithm (ImageScope) on scanned slides. The number of TLR4+ macrophages and CD42b+ platelets was counted in high‐powered fields (HPF, 40× magnification).

For each analysis, at least 10 regions of interest (i.e., HPFs or portal tracts) per biopsy were examined and counted. The presence of at least 10 regions of interest in a specific stain was considered a minimal requirement for its evaluation. When a stained sample did not meet this requirement, it was excluded from the specific analysis.

### 2.3 Statistical Analysis

Data were expressed as median (interquartile range) or means ± standard deviation. Normality was assessed by Shapiro–Wilk normality test; appropriate tests were chosen accordingly.

Comparison between groups was performed by unpaired Student t test or Mann–Whitney *U*‐test, when appropriate, by analysis of variance (ANOVA) and by Newman–Keuls multiple comparison or by Kruskal–Wallis and Dunn's multiple comparisons test *post hoc* analyses. Differences in dichotomous variables were assessed by chi‐square test. Correlation was assessed by Pearson's correlation coefficient or by Spearman's rank correlation coefficient. *p*‐value of less than 0.05 was considered statistically significant. Stepwise linear regression analysis was performed to individuate independently correlated variables. The Number Cruncher Statistical System (NCSS), version R3.4.0, and IBM SPSS Statistics (v. 27.0.1.0, IMB corp., Armonk, NY, USA) were used.

## 3 Results

Demographical, clinical and biochemical characteristics of the patients included in the study are shown in Table 1.

**TABLE 1:** Demographical and biochemical characteristics of the 17 patients with PSVD without portal hypertension (PSVD PH−), of the 17 patients with PSVD with portal hypertension (PSVD PH+) and of 17 healthy controls included in the study.

	PSVD PH−(*n* = 17)	PSVD PH+(*n* = 17)	Healthy subjects (*n* = 17)	*p*
Age (years)	42 (22–58)	51 (20–68)	48 (20–58)	0.2
Sex (female)	9 (53%)	4 (24%)	8 (47%)	0.1
Bilirubin (mg/dL)	0.9 (0.3–1.4)	1.2 (0.8–1.8)	0.4 (0.2–0.7)	< 0.001
AST (U/L)	39 (14–85)	29 (15–86)	26 (13–38)	0.01
ALT (U/L)	80 (15–199)	27 (10–305)	27 (6–48)	0.006
Alkaline phosphatase (AP) (U/L)	108 (55–169)	100 (34–403)	89 (62–129)	0.3
γ‐glutamyltransferase (γ‐GT) (U/L)	148 (63–447)	77 (16–178)	28 (13–46)	< 0.001
Albumin (g/dL)	4.1 (3.7–4.7)	3.9 (3–4.3)	3.8 (3.3–4‐6)	0.013
INR	1 (0.6–1.2)	1.1 (0.9–1.3)	1 (0.7–1)	0.06
Leucocytes (×10^3^/μL)	7 (6.2–7.6)	5 (4.2–3.8)	6.8 (5.9–7.8)	0.02
Neutrophils (×10^3^/μL)	4.1 (3.7–4.4)	3 (2.9–4)	4 (3.3–4.6)	0.2
C reactive protein (mg/L)	3.9 (2.9–4.7)	4 (3.1–5)	3 (2.3–3.7)	0.03
Platelets (×10^3^/μL)	238 (162–325)	136 (47–229)	207 (153–245)	< 0.001
Thrombocytopenia (yes)	0	12 (70%)	0	< 0.001
Splenomegaly (yes)	0	13 (76%)	0	< 0.001
Oesophagogastric varices (absent/small/large)	17 (100%)/0/0	7 (41%)/4 (26%)/6 (35%)	17 (100%)/0/0	< 0.001
Gastric varices (yes)	0	1 (6%)	0	0.3
Portal‐hypertensive bleeding (yes)	0	6 (35%)	0	< 0.001
Endoscopic bending ligation (yes)	0	6 (35%)	0	< 0.001
Ascites (yes)	0	3 (18%)	0	0.04
Portosystemic shunt (yes)	0	0	0	
Liver stiffness (kPa)^a^	5.1 (3.7–7.3)	5.5 (2.4–10)	/	0.2

*Note:* For continuous variables, data are expressed as median (IQR). For non‐continuous variables, data are expressed as *N* (%).

Abbreviations: γ‐GT, γ‐glutamyl transferase; ALT, alanine aminotransferase; AP, alkaline phosphatase; AST, aspartate aminotransferase; INR, international normalised ratio.

^a^ Not available in one patient's PSVD PH− and in two patients PSVD PH+.

All patients with PSVD without PH had normal liver synthetic function and normal platelet count, but chronic elevation of liver enzymes. Specifically, biopsy was performed for the elevation of aspartate aminotransferase and/or alanine aminotransferase in one patient, for elevated γ‐glutamyl transferase in four patients, and for the alteration in both transaminases and γ‐glutamyl transferase in 12 patients. Many of these patients (41%) had a condition associated with PSVD, mostly represented by immune system disorders (Table 2). One patient had celiac disease, six patients had an autoimmune disorder, and one of these patients had multiple sclerosis. In none of the patients with PSVD without PH was liver disease secondary to the use of drugs such as oxaliplatin or azathioprine, but one patient was a chronic user of estroprogestins.

**TABLE 2:** Conditions associated with porto‐sinusoidal vascular disorder (PSVD) in the 34 patients included in the study.

	PSVD PH+ (*n* = 17)	PSVD PH− (*n* = 17)
Associate disorder (No/Yes)	8 (47%)/9 (53%)	10 (59%)/7 (41%)
Acquired and congenital immunodeficiency		
Primary antibody‐deficiency syndrome	1 (6%)	0
HIV chronic infection	1 (6%)	0
Autoimmune diseases		
Systemic sclerosis	1 (6%)	1 (6%)
Coeliac disease	0	1 (6%)
Autoimmune hepatitis	1 (6%)	0
Hashimoto's thyroiditis	0	4 (24%)
Multiple sclerosis	0	1 (6%)
Genetic disorders		
Cystic fibrosis	1 (6%)	0
Drugs		
Oxaliplatin	1 (6%)	0
HAART	1 (6%)	0
Thrombophilia		
Protein C deficiency	1 (6%)	0
Protein S deficiency	1 (6%)	0
Lupus anticoagulant (LAC)	2 (12%)	0
Other haematological disorders		
Plasmacytoma	0	1 (6%)

*Note:* For non‐continuous variables, data are expressed as *N* (%).

Abbreviations: HAART, highly active anti‐retroviral therapy; HIV, human immunodeficiency virus; LAC, lupus anticoagulant.

None of the patients had PSVD in combination with alcoholic liver disease, chronic viral hepatitis, or moderate‐to‐severe metabolic dysfunction‐associated steatotic liver disease. Only one patient in the PSVD without PH group and two in the PSVD with PH group had mild metabolic dysfunction‐associated steatotic liver disease.

Patients of the three groups did not show significant alterations of systemic inflammatory markers.

According to the inclusion criteria, none of the patients with PSVD without portal hypertension (PH−) had specific or non‐specific signs of portal hypertension, while in the control group most patients had splenomegaly (76%) and esophago‐gastric varices (58%); 35% of the patients had previously experienced portal‐hypertensive bleeding (Table 1) and all of them were submitted to combined therapy with non‐selective beta‐blockers and endoscopic variceal banding ligation. Only 3 patients had ascites requiring low dosage of diuretics. Two patients were on anticoagulants for the treatment of portal vein thrombosis sustained by a prothrombotic condition. None of the patients had portosystemic shunts.

Results of the comparison of serological markers of intestinal permeability and of bacterial translocation, platelet aggregation and hypercoagulability between patients with PSVD without PH and a group of healthy subjects matched for age and sex, are shown in Table 3. Patients with PSVD PH− had higher levels of zonulin (2.15 ± 0.8 vs. 1.36 ± 0.5 ng/mL, *p* < 0.0001) and LPS (26 ± 10 vs. 20 ± 3 pg/mL, *p* < 0.0001) and significantly higher mean levels of sP‐selectin (24 ± 11 vs. 17 ± 7 ng/mL, *p* = 0.03) and sGPVI (36 ± 15 vs. 27 ± 9 pg/mL, *p* = 0.04), demonstrating an increased pro‐aggregation state.

**TABLE 3:** Comparison of serum zonulin, lipopolysaccharides, markers of platelet aggregation and of hypercoagulability between patients with PSVD without portal hypertension (PSVD PH−) and healthy controls.

	PSVD PH− (*n* = 17)	Healthy controls (*n* = 17)	*p*
Zonulin (ng/mL)	2.15 ± 0.87	1.36 ± 0.51	0.0035
LPS pg/mL	26 ± 10	20 ± 3	0.02
vWF (U/dL)	203 ± 52	169 ± 48	0.05
ADAMTS13 (ng/mL)	338 ± 72	387 ± 69	0.049
sGP VI (pg/mL)	36 ± 15	27 ± 9	0.04
sP‐selectin (ng/mL)	24 ± 11	17 ± 7	0.03
Factor VIII (U/dL)	117 ± 24	89 ± 23	0.0016
F1 + 2 (pmol/L)	156 ± 37	131 ± 25	0.029

Abbreviations: F1 + 2, prothrombin fragment F1 + 2; LPS, lipopolysaccharides; sGPVI, soluble glycoprotein VI; sPs, soluble p‐selectin; vWF, von Willebrand factor.

The results showed that PSVD PH− patients had decreased ADAMTS13 activity (338 ± 72 vs. 387 ± 69 ng/mL, *p* = 0.049) and almost significantly higher vWF levels (203 ± 52 vs. 169 ± 48 U/dL, *p* = 0.05) compared to healthy subjects, denoting endothelial damage. Again, PSVD PH− showed increased levels of factor VIII (117 ± 24 vs. 89 ± 23 U/dL, *p* = 0.0016) and F1 + 2 (156 ± 37 vs. 131 ± 25 pmol/L, *p* = 0.029) indicating pro‐coagulant platelet activation.

**FIGURE 1:**
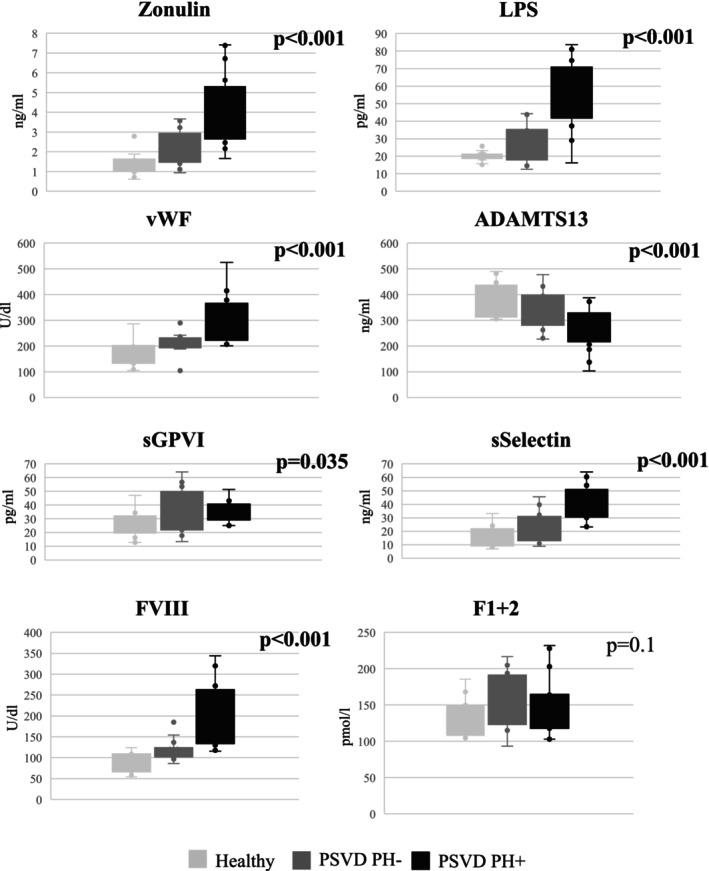
Comparison of serum zonulin, lipopolysaccharides, markers of platelet aggregation and of hypercoagulability between patients with PSVD without portal hypertension (PSVD PH−), patients with PSVD with portal hypertension (PSVD PH+) and healthy controls.

Finally, we compared the same markers in patients with PSVD without portal hypertension, patients with PSVD with PH+ and healthy subjects (Figure 1). Interestingly, zonulin, LPS, as well as markers of platelet aggregation and hypercoagulability showed a progressive increase among the three groups, being elevated in patients with PSVD PH− and even higher in those with PSVD PH+. In contrast, F1 + 2 levels were similar between the three groups.

### 3.1 Histopathological Examination of Liver Biopsies

After a preliminary evaluation, *N* = 17 biopsies from patients with PSVD and PH and *N* = 17 biopsies from those with PSVD without PH were considered adequate and included in subsequent analyses. As histologic controls, *N* = 6 liver samples from liver donors were used.

**FIGURE 2:**
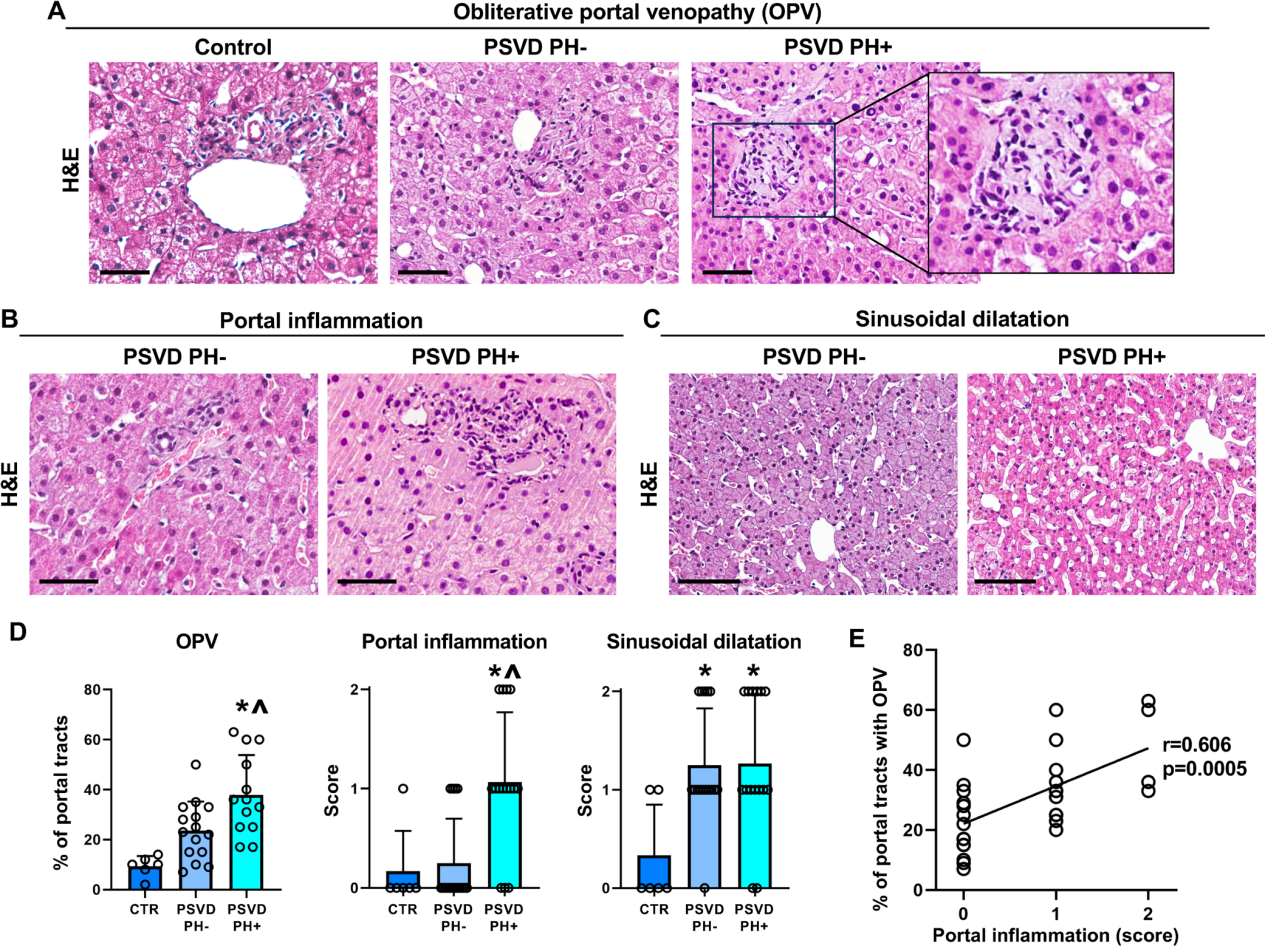
Histomorphological features in PSVD with or without portal hypertension (PH). (A–C) Haematoxylin and eosin (H&E) stain in control liver samples and in liver biopsies obtained from people with PSVD with or without portal hypertension (PH+ and PH−). Patients with PSVD were characterised by obliterative portal venopathy (OPV) (A), portal inflammation (B) and sinusoid dilation (C). Area in the box in A is magnified to the right. Scale bars: 50 μm (A and B) and 100 μm (C). (D) Graphs show means and standard deviation for the percentage of portal tracts with OPV, portal inflammation score, and sinusoid dilation score in healthy livers (controls, CTR) and in liver biopsies obtained from people with PSVD with PH or without PH. *p < 0.05 vs. CTR; ^*p* < 0.05 vs. CTR and PSVD PH−. (E) Scatter plot shows correlation between OPV and portal inflammation score in all PSVD samples.

In the studied cohort, all biopsies from people with PSVD showed signs of OPV (Figure 2A,D). When compared to healthy liver controls, the percentage of portal tracts with OPV was significantly higher in patients with PSVD PH+ (*p* = 0.0002) but not in those PH− (*p* = 0.066). Moreover, patients with PH showed significantly more frequent OPV compared to those without PH (*p* = 0.012). When we assessed inflammatory infiltration, we observed that portal inflammation (Figure 2B,D) was significantly more prominent in PSVD patients with signs of portal hypertension compared to those PH− (*p* = 0.003) or controls (*p* = 0.019). No significant difference in inflammatory infiltration was observed between patients with PSVD PH− compared to controls. When we evaluated alterations affecting liver sinusoids (Figure 2C,D), PSVD patients showed more prominent sinusoidal dilation compared to normal livers (*p* = 0.024 for PSVD PH− and *p* = 0.020 for PSVD PH+). No significant differences were observed among PSVD patients based on the presence/absence of PH.

When the relationship between OPV and other histological features was investigated, portal inflammation but not sinusoidal dilation was significantly correlated with the percentage of portal tracts affected by OPV (*r* = 0.606; *p* = 0.0005; Figure 2E). Moreover, when histological features were correlated with clinical parameters in patients with PSVD, we observed that OPV correlated with LPS (*r* = 0.435; *p* = 0.034) and FVIII (*r* = 0.585; *p* = 0.003) serum levels, and with liver stiffness (*r* = 0.414; *p* = 0.036; Figure S1A); at multivariate regression analysis, only FVIII was independently correlated with OPV (beta = 0.592; *p* = 0.003). Furthermore, portal inflammation correlated with zonulin (*r* = 0.501; *p* = 0.008), vWF (*r* = 0.452; *p* = 0.020) and FVIII (*r* = 0.466; *p* = 0.016) serum levels. In multivariate regression analysis, only zonulin resulted independently correlated with portal inflammation (beta = 0.493; *p* = 0.012).

**FIGURE 3:**
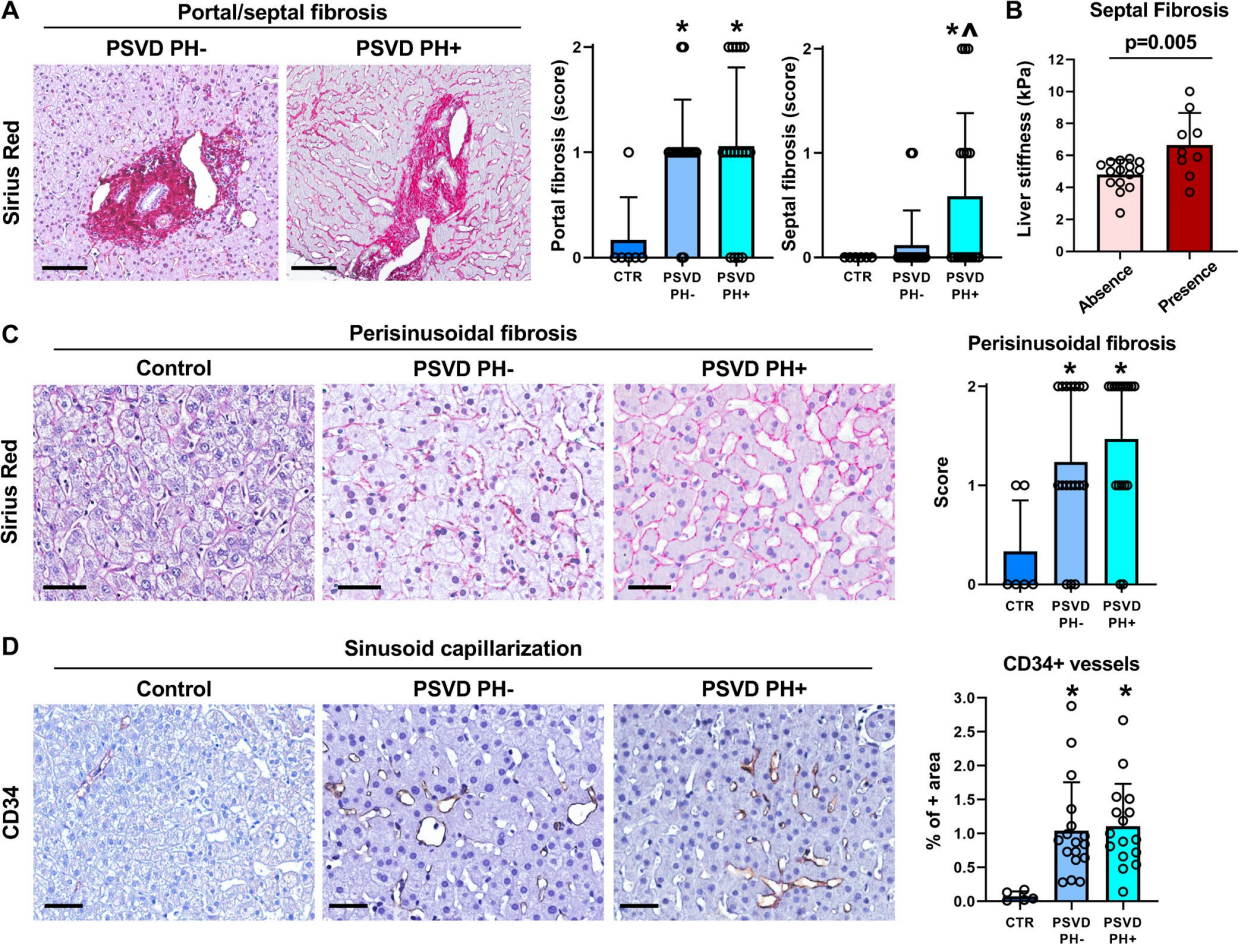
Fibrosis and sinusoid capillarisation in PSVD with or without portal hypertension (PH). (A) Sirius Red stain in liver biopsies obtained from people with PSVD with or without portal hypertension (PH+ and PH−). Patients with PSVD with PH showed higher portal and septal fibrosis. Scale bars: 100 μm. Graphs report means and standard deviation for portal and septal fibrosis scores in controls (CTR) and in biopsies obtained from people with PSVD with PH or without PH. *p < 0.05 vs. CTR; ^*p* < 0.05 vs. CTR and PSVD PH−. (B) Graph shows means and standard deviation for liver stiffness values in PSVD patients based on the presence or absence of septal fibrosis at liver biopsies. (C) Sirius Red stain in control samples and in liver biopsies obtained from people with PSVD with or without portal hypertension. Scale bars: 50 μm. Graph reports means and standard deviation for perisinusoidal fibrosis scores in controls and in biopsies obtained from people with PSVD with PH or without PH. *p < 0.05 vs. CTR. (D) Immunohistochemistry for CD34 in control samples and in biopsies obtained from people with PSVD with or without PH. PSVD patients showed significantly higher sinusoid capillarisation compared to controls. Scale bars: 50 μm. Graph reports means and standard deviations for the percentage of liver area occupied by CD34+ vessels in controls and in biopsies obtained from people with PSVD with PH or without PH. *p < 0.05 vs. CTR.

The degree of fibrosis is shown in Figure 3A–C. PSVD patients were characterised by significantly more prominent portal fibrosis compared to controls (*p* = 0.020 for PSVD PH− and *p* = 0.014 for PSVD PH+). When septal fibrosis was evaluated, it was significantly higher in PSVD patients with PH (*p* = 0.038 vs. controls) but not in those without PH. Perisinusoidal fibrosis was significantly higher in PSVD patients compared to controls (*p* = 0.020 for PSVD PH− and *p* = 0.003 for PSVD PH+). The overall fibrosis score was also significantly higher in PSVD patients compared to controls (*p* = 0.006 for PSVD PH− and *p* = 0.0005 for PSVD PH+). As expected, no case had a fibrosis score = 4 (i.e., cirrhosis); only *N* = 3 cases (all in the PH+ group) showed advanced fibrosis (i.e., fibrosis score = 3). No differences were observed in terms of portal and perisinusoidal fibrosis, and of overall fibrosis score between PSVD patients with and without PH. Septal fibrosis, however, was significantly higher in patients with PH compared to those without (*p* = 0.033).

When the relationship between fibrosis and histological features of PSVD was assessed, both septal fibrosis and overall fibrosis score correlated with OPV (*r* = 0.580 and *r* = 0.385, respectively; *p* = 0.002 and *p* = 0.043; Figure S1B) and portal inflammation (*r* = 0.509 and *r* = 0.535, respectively; *p* = 0.005 and *p* = 0.002); no correlation was observed between fibrosis scores and sinusoidal dilation. When stepwise linear regression analysis was performed, we observed that septal fibrosis, as a dependent variable, was independently correlated with portal inflammation (beta = 0.472; *p* = 0.007) and portal fibrosis (beta = 0.485; *p* < 0.001). In a similar model, the percentage of portal tracts affected by OPV was independently correlated with portal inflammation (beta = 0.613; *p* < 0.01), but not with the other histological parameters (i.e., sinusoidal dilatation, perisinusoidal fibrosis, portal fibrosis and septal fibrosis).

When fibrosis scores were correlated with serological parameters, portal fibrosis inversely correlated with sP‐selectin (*r* = −0.447; *p* = 0.017), and overall fibrosis score correlated with FVIII levels (*r* = 0.388; *p* = 0.041). Interestingly, patients with septal fibrosis were characterised by higher liver stiffness compared to those without (*p* = 0.005; Figure 3B), and septal fibrosis correlated with stiffness values (*r* = 0.427; *p* = 0.027; Figure S1C), also independently of other serological investigated biomarkers at multivariate regression analysis (beta = 0.499; *p* = 0.009).

To complete sinusoid modification assessment, we studied sinusoid capillarisation (i.e., extent of CD34+ endothelium in sinusoid). Interestingly, sinusoid capillarisation (Figure 3D) was significantly more prominent in patients with PSVD compared to controls (*p* = 0.014 for PSVD PH− and *p* = 0.009 for PSVD PH+). No significant differences were observed among PSVD patients based on the presence/absence of PH. When we investigated the pattern of sinusoid capillarisation (Table S3), all PSVD patients showed involvement of hepatic sinusoids around and between portal tracts (zone 1 of liver lobule). Capillarisation was limited to zone 1 more frequently in PSVD PH− patients (*p* = 0.015 vs. PSVD PH+), while it showed a panlobular pattern only in PSVD PH+ (*p* = 0.007 vs. PSVD PH−). In our cohort, capillarisation limited to pericentral areas was never observed.

### 3.2 TLR4+ Macrophages and Platelets in Liver Biopsies

**FIGURE 4:**
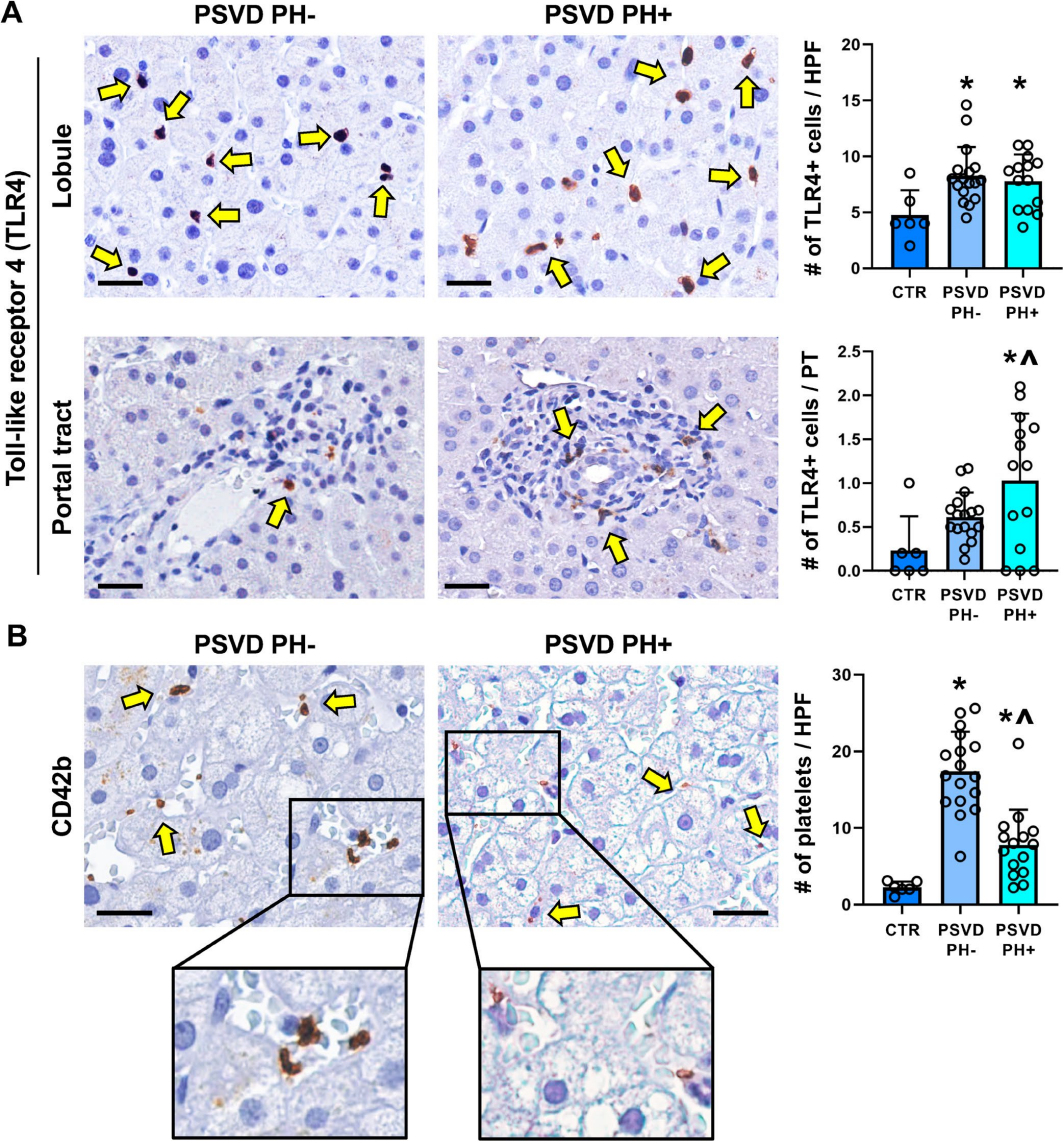
Pro‐inflammatory cells and platelets in PSVD with or without portal hypertension (PH). (A) Immunohistochemistry for toll‐like receptor factor 4 (TLR4) in PSVD in liver biopsies obtained from people with PSVD with or without portal hypertension (PH+ and PH−). PSVD patients were characterised by pro‐inflammatory TLR4+ cells (arrows) within liver lobules (upper panels) and portal tracts (lower panels). Scale bars: 25 μm. Graphs report means and standard deviation for the number of TLR4+ cells per high‐powered field (HPF) in liver lobules and in portal tracts (PT) in healthy livers (controls, CTR) and in liver biopsies obtained from people with PSVD with PH or without PH. *p < 0.05 vs. CTR; ^*p* < 0.05 vs. CTR and PSVD PH−. (B) Immunohistochemistry for CD42b in liver biopsies obtained from people with PSVD with or without portal hypertension (PH+ and PH−). PSVD patients were characterised by CD42b + platelets (arrows) within liver lobules. Areas in the box are magnified below. Scale bars: 25 μm. Graphs report means and standard deviation for the number of CD42+ platelets (PLT) per HPF in liver lobules in healthy livers and in liver biopsies obtained from people with PSVD with PH or without PH. *p < 0.05 vs. CTR; ^*p* < 0.05 vs. CTR and PSVD PH−.

When the number of TLR4+ macrophages was assessed (Figure 4A), PSVD patients showed a higher number of TLR4+ macrophages within the liver lobule (PSVD PH−: 7.9 [6.5–8.9] positive cells/HPF, *p* = 0.014; PSVD PH+: 8.3 [5.2–9.6] positive cells/HPF, *p* = 0.044) compared to normal livers (4.0 [3.5–6.6] positive cells/HPF). Moreover, a higher number of TLR4+ cells within the portal tracts was observed in PSVD with PH (1.2 [0.2–1.7] positive cells/HPF, *p* = 0.010), but not in those without PH (0.6 [0.5–0.7] positive cells/HPF), compared to controls (0.1 [0–0.7] positive cells/HPF). PSVD patients with PH displayed a significantly higher number of TLR4+ cells within the portal tracts (*p* = 0.037) but not within liver parenchyma compared to PSVD patients without PH. Interestingly, the number of portal TLR4+ cells correlated with the portal inflammation score (*r* = 0.447; *p* = 0.017), fibrosis scores (portal fibrosis: *r* = 0.360, *p* = 0.047; septal fibrosis: *r* = 0.427, *p* = 0.017; overall fibrosis score: *r* = 0.371, *p* = 0.040).

Finally, we evaluated the number of (CD42+) platelets within liver sinusoids (Figure 4B). PSVD patients showed a higher number of platelets within sinusoids compared to normal livers (PSVD PH−: 16.8 [13.5–21.4] platelets/HPF, *p* < 0.0001; PSVD PH+: 7.9 [4.2–9.6] platelets/HPF, *p* = 0.042) and signs of aggregation, such as clumping and endothelium adhesion (*p* = 0.0009 for PSVD PH− and *p* = 0.030 for PSVD PH+). Interestingly, platelets were significantly more numerous in sinusoids of PSVD patients without PH compared to those with PH (*p* < 0.0001), without significant differences in terms of signs of aggregation. Finally, the number of platelets correlated with lobular TLR4+ cells (*r* = 0.371; *p* = 0.043).

### 3.3 Follow‐Up Evaluation

During a median follow‐up of 28 months (range 14–40 months), none of the 17 PSVD PH− patients developed clinical, biochemical, or radiological signs suggestive of PH. NoPH‐related complications occurred, such as gastrointestinal bleeding or thrombosis of the spleno‐portal axis. Regular monitoring, including abdominal ultrasound with Doppler and routine blood tests every 6–12 months, revealed no new onset of splenomegaly, ascites, or thrombocytopenia.

## 4 Discussion

PSVD is a condition histologically characterised by lesions involving portal veins and sinusoids in the absence of cirrhosis [1]. Clinically, it can occur in the absence of evident PH, often associated with mild to moderate elevations in liver enzymes, or in the presence of signs of PH [1, 5, 6, 7]. The aetiology and pathogenesis of PSVD remain poorly understood.

In the present study, we explored the potential role of gut‐derived endotoxins in the pathogenesis of PSVD by analysing a cohort of patients affected by PSVD PH− in comparison with a group of healthy subjects and a group of patients affected by PSVD and PSVD PH+.

The first relevant result of the study was that in the 17 patients with PSVD PH−, biochemical and histological changes similar to those previously demonstrated [14] in PSVD PH+ were observed. Specifically, serum levels of zonulin and LPS were significantly higher in PSVD PH− compared to healthy subjects, confirming, by measuring indirect biomarkers, the presence of intestinal barrier integrity alterations and bacterial translocation in PSVD, even in the absence of PH.

The markers of endothelial damage (reduced ADAMTS13 and elevated vWF), of platelet aggregation (sp‐Selectin and GPVI) and those of coagulation cascade activation (FVIII, F1 + 2) were significantly altered in PSVD patients without PH compared to healthy controls.

In our series, these serological alterations are paralleled by morphologic changes within sinusoids observed in liver biopsies; sinusoids appeared dilated and showed aspects of capillarisation (CD34 positivity in endothelial cells) and evidence of perisinusoidal fibrosis when compared to control samples. Remarkably, platelets within sinusoids were significantly increased in patients with PSVD PH than in control subjects, showing signs of aggregation, such as clumping and endothelium adhesion.

These results support the putative role of the gut‐derived endotoxins in the instauration of liver endothelial damage and the involvement of a pro‐aggregating and pro‐coagulant state in the pathogenesis of the PSVD. This hypothesis is strengthened by the fact that the number of TLR4+ macrophages, which is a selective binder of LPS, was significantly increased in liver biopsies of patients with PSVD without PH compared to healthy controls. Again, the results suggest the activation of the LPS‐TLR4 pathway in patients affected by PSVD, with and without PH. These results are similar to those observed in cirrhotic patients in whom higher serum levels of LPS as well as factor VIII and vWF, than the control group have been documented [17]. However, contrary to cirrhosis, our results may support the idea that the LPS‐induced hypercoagulability may represent the pathophysiological basis of the vascular lesions of PSVD.

The second relevant result of the study is the evidence of a progression of the disease, transitioning from the PSVD PH− to the PSVD PH+. In fact, serological markers of intestinal permeability and bacterial translocation, as well as markers of endothelial damage, platelet aggregation, and hypercoagulability were higher in patients PH− compared to healthy controls; however, these alterations were even more pronounced in PSVD PH+ patients (Figure 1). This observation may reflect the different severity of the disease, where PSVD PH− may represent an early and subclinical stage of PSVD, while PSVD PH+ may represent a full‐blown stage with significant liver disease. Moreover, the occurrence of PH in PSVD may exacerbate gut barrier alterations by determining oedema within the intestinal mucosa; this could further favour bacterial translocation by direct alteration of tight junctions between the intestinal epithelial cells [18].

The presence of a progression from PSVD PH− to PH+ can also be hypothesised based on histological liver alterations. PSVD PH+ patients were characterised by more prominent OPV and portal inflammation compared to PSVD PH− patients. In PSVD PH−, TLR4+ cells were predominantly located in liver lobules, while in PH+, TLR4+ macrophages were located both in liver lobules and in the portal tract. On the contrary, sinusoid alterations were higher in PSVD PH− than in controls but equal to PSVD PH+. Sinusoid capillarisation appeared more often limited to zone 1 in PSVD PH− patients compared to those PH+, thus suggesting a progressive extension of this alteration toward central veins in the advanced disease phase. Moreover, the number of platelets in the liver sinusoids, likely secondary to the hyperexpression of markers of platelet aggregation, was very high in patients PH−, even more than in PH+. These observations suggest that in patients PSVD PH−, vascular damage is primarily localised at the sinusoidal level rather than at the portal veins. Only some patients with PSVD PH− showed features of OPV and portal inflammation; on the contrary, patients PSVD PH+, in addition to the sinusoidal involvement, very frequently demonstrated OPV associated with severe portal inflammation. Interestingly, OPV was correlated with portal inflammation and the development of fibrosis, particularly with septal fibrosis, a specific hallmark of PSVD. In our cohort, septal fibrosis correlated with liver stiffness; although in a small cohort, this preliminary observation could open future studies for developing the use of elastography in the clinical management of these patients.

We can speculate that in PSVD, the damage starts at the sinusoidal level as a consequence of a triggering stimulus. Our hypothesis is that these stimuli are represented by gut‐derived endotoxins. In fact, the TLR‐4 cells, responsible for LPS binding, act initially in the sinusoid, promoting platelet activation and adhesion at the vascular endothelium, creating a pro‐inflammatory environment. Successively, probably due to the persistence of stimuli or for other unknown reasons, the damage extends from the sinusoids to the portal tract, involving the portal veins. Platelet adhesion, inflammation, and fibrosis may lead to portal vein sclerosis (OPV) and to the development of PH.

In summary, this study showed that even before the development of clinically evident PH, patients with a histological diagnosis of PSVD already exhibit alterations in gut barrier integrity, increased translocation of bacteria‐derived particles, and related consequences such as platelet aggregation and hypercoagulability. These findings suggest that endotoxemia, resulting from altered intestinal permeability, could play a key role in the pathogenesis of vascular alterations underlying PSVD. Moreover, for the first time, this study provides evidence that PSVD without and with PH may represent different stages of the same disease. PSVD without PH could represent an early stage of the disease that may progress to a clinically more advanced stage represented by PSVD with PH.

This study has some limitations, including the small sample size that limits the generalisability of the findings and underscores the need for future longitudinal studies with larger cohorts to confirm and expand upon these observations. A larger sample would strengthen the robustness of the results. However, it is important to emphasise that PSVD is a rare condition, and among affected individuals, those without PH—our study's focus—represent a minority. Furthermore, we want to highlight that the topic addressed in our study remains largely unexplored in the existing literature. As such, our work represents an initial step towards filling this gap and offers novel insights that we believe are valuable, even at this preliminary stage.

Moreover, the current inflammatory profiling, while focused and hypothesis‐driven, may represent a relatively limited and targeted strategy, which may not capture the full complexity of the inflammatory landscape involved in PSVD. We aimed to specifically test the hypothesis of a role for bacterial translocation and low‐grade systemic inflammation, which our findings appear to support. More comprehensive and unbiased approaches, such as single‐cell transcriptomics and spatially resolved technologies, may hold great potential to unravel the complexity of PSVD pathogenesis. These methodologies could provide deeper insights into the tissue‐specific and cell type‐specific processes involved, including previously unrecognised inflammatory or immune pathways.

Despite these limitations, the study lays a foundation for future research, such as the identification of PSVD‐related gut microbiome. Furthermore, these results may have many therapeutic implications, suggesting that interventions targeting the gut‐liver axis could be considered in the management of PSVD. To date, as in cirrhosis, therapeutic options are limited to agents addressing complications related to PH and portal vein thrombosis. If confirmed in further studies, our results may open the possibility of trials focused on the use of drugs acting on the reduction of endotoxemia or intestinal permeability as well as antiaggregant or anticoagulants for the vascular inflammation, even at the early‐stage PSVD.

## Author Contributions

S.G., D.O., N.P., R.C., A.A., O.R., G.C.: study concept and design, acquisition of data, analysis and interpretation of data, drafting of the manuscript, critical revision of the manuscript for important intellectual content, statistical analysis and study supervision. S.N., D.T., L.R., A.M., M.M., L.d.T., L.T., G.d.A., A.B., M.F., G.F., E.G.: acquisition of data, analysis and interpretation of data, drafting of the manuscript, critical revision of the manuscript for important intellectual content.

## Ethics Statement

The study was conducted in accordance with the ethical principles of the Declaration of Helsinki and the institutional review board of the Sapienza University of Rome (Prot no. 0407/2022, ref. 6713) and of Humanitas Research Hospital (Prot no. 155/23) approved the study protocol.

## Consent

Written informed consent was obtained from each patient.

## Conflicts of Interest

The authors declare no conflicts of interest.

## Supporting information


**Table S1:** Primary antibodies for immunohistochemistry.
**Table S2:** Histological scoring of liver biopsies.
**Table S3:** Pattern of sinusoid capillarisation in patients with PSVD without portal hypertension (PH−) and with portal hypertension (PH+).

**Figure S1:** Scatter plots show correlation analyses, in all PSVD samples, between: (A) OPV and liver stiffness; (B) OPV and septal fibrosis score; (C) liver stiffness and septal fibrosis score.

**Data S1:** liv70277‐sup‐0003‐DataS1.docx.

## Data Availability

The data that support the findings of this study are available from the corresponding author upon reasonable request.
