# Supplementary material for: Role of Gut‐Derived Endotoxins in Porto‐Sinusoidal Vascular Disorder: Comparison Between patients with and without portal hypertension
Source: Liver Int. 2025 Aug 8;45(9):e70277. doi: 10.1111/liv.70277 (PMC12334859; doi:10.1111/liv.70277)
Supplement: Supplementary file 1 — Table S1: Primary antibodies for immunohistochemistry. Table S2: Histological scoring of liver biopsies. Table S3: Pattern of sinusoid capillarisation in patients with PSVD without portal hypertension (PH−) and with portal hypertension (PH+). [file LIV-45-0-s002.docx]

**SUPPLEMENTARY TABLES.**

**Supplementary Table 1. Primary antibodies for immunohistochemistry.**

| **Antibody** | **Host species** | **Manufacturer** | **Code** | **Clone** | **Dilution** |
| --- | --- | --- | --- | --- | --- |
| CD34 | Mouse | Dako | M7165 | QBEnd 10 | 1:50 |
| CD42b | Rabbit | abcam | ab183345 | SP219 | 1:100 |
| TLR4 | Rabbit | Atlas Antibodies | HPA049174 | polyclonal | 1:200 |

**List of abbreviations**. CD: cluster of differentiation; TLR: toll-like receptor.

**Manufacturers**. *Abcam*, Cambridge, United Kingdom; *Atlas Antibodies*, Bromma, Sweden; *Dako (Agilent)*, Glostrup, Denmark.

**Supplementary Table 2. Histological scoring of liver biopsies.**

| **Item** | **Semi-quantitative score** |
| --- | --- |
| Sinusoidal dilatation | 0= <5% of sinusoids  1= 5-30% of sinusoids  2= >30% of sinusoids |
| Lobular inflammation | 0= no foci  1= 2-3 foci per 20x field  2= > 3 foci per 20x field |
| Perisinusoidal fibrosis | 0= no fibrosis  1= 5-30% of sinusoids  2= > 30% of sinusoids |
| Portal inflammation | 0= none  1= < 30 % of portal tracts  2= > 30% of portal tracts |
| Fibrosis | 0= no fibrosis  1= perisinusoidal or periportal  2= perisinusoidal and periportal  3= septal/bridging  4= cirrhosis |

**Supplementary Table 3. Pattern of sinusoid capillarization in patients with PSVD without portal hypertension (PH-) and with portal hypertension (PH+).**

| **Overall pattern of sinusoid capillarization** | **PSVD PH-**  **(N=17)** | **PSVD PH+**  **(N=17)** | **p-value** |
| --- | --- | --- | --- |
| **Zone 1 only** | 13/17 (76.5%) | 5/17 (29.4%) | 0.015 |
| **Zone 2 or 3 only** | 0/17 | 0/17 | ns |
| **Zone 1 and 3** | 4/17 (23.5%) | 5/17 (29.4%) | ns |
| **Panlobular** | 0/17 | 7/17 (41.2%) | 0.007 |

For non-continuous variables, data are expressed as N (%).
