# Supplementary figures and images for: Role of Gut‐Derived Endotoxins in Porto‐Sinusoidal Vascular Disorder: Comparison Between patients with and without portal hypertension
Source: Liver Int. 2025 Aug 8;45(9):e70277. doi: 10.1111/liv.70277 (PMC12334859; doi:10.1111/liv.70277)

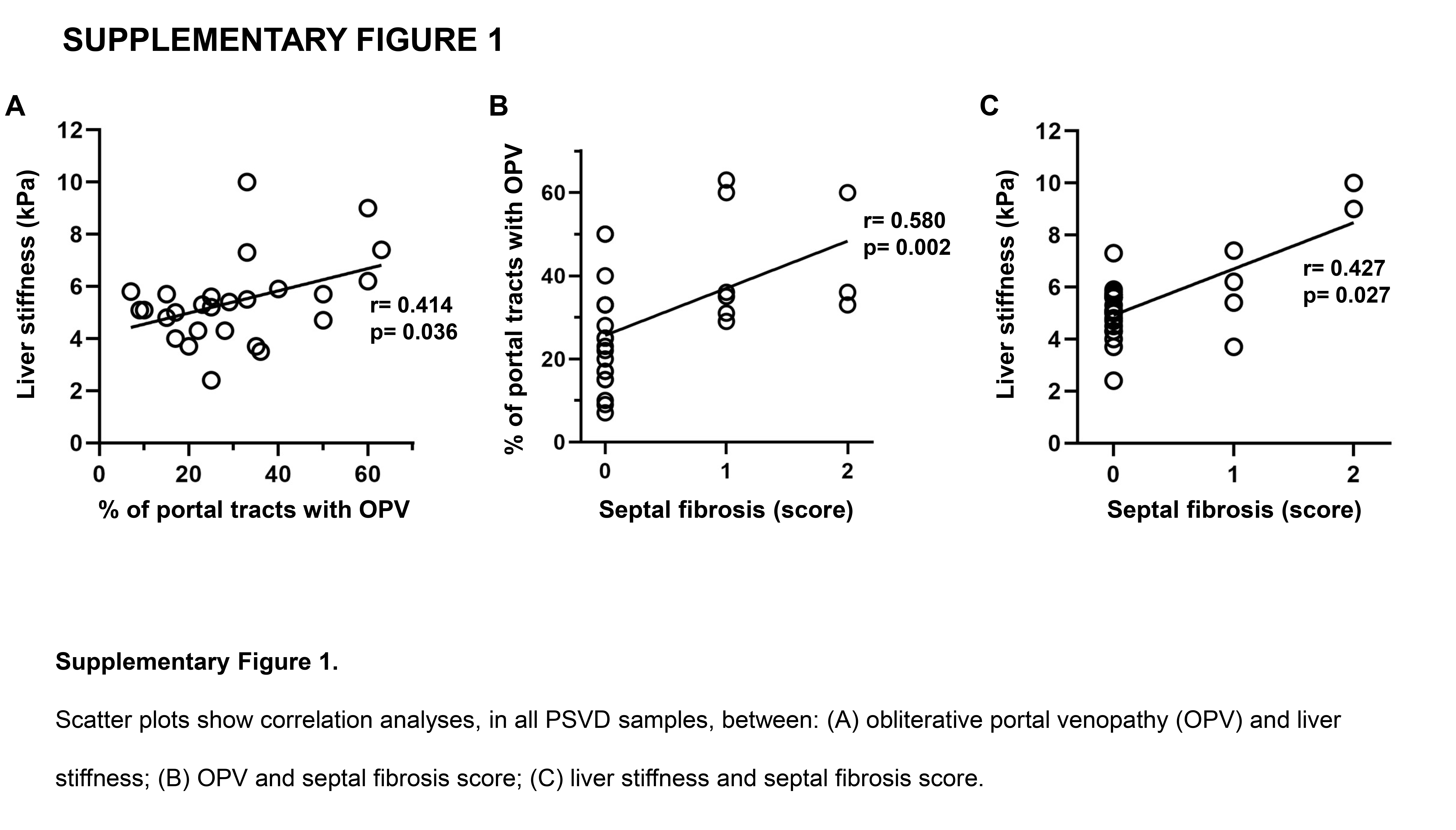

Supplement: Supplementary file 2 — Figure S1: Scatter plots show correlation analyses, in all PSVD samples, between: (A) OPV and liver stiffness; (B) OPV and septal fibrosis score; (C) liver stiffness and septal fibrosis score. [file LIV-45-0-s003.tif]
