# Supplementary material for: Role of Gut‐Derived Endotoxins in Porto‐Sinusoidal Vascular Disorder: Comparison Between patients with and without portal hypertension
Source: Liver Int. 2025 Aug 8;45(9):e70277. doi: 10.1111/liv.70277 (PMC12334859; doi:10.1111/liv.70277)
Supplement: Supplementary file 3 — Data S1: liv70277‐sup‐0003‐DataS1.docx. [file LIV-45-0-s001.docx]

**SUPPLEMENTARY FIGURE LEGEND**

**Supplementary Figure 1.**

Scatter plots show correlation analyses, in all PSVD samples, between: (A) OPV and liver stiffness; (B) OPV and septal fibrosis score; (C) liver stiffness and septal fibrosis score.
